# SEOM-GEMCAD-TTD clinical guidelines for the systemic treatment of metastatic colorectal cancer (2022)

**DOI:** 10.1007/s12094-023-03199-1

**Published:** 2023-05-03

**Authors:** Ana Fernández Montes, Vicente Alonso, Enrique Aranda, Elena Élez, Pilar García Alfonso, Cristina Grávalos, Joan Maurel, Ruth Vera, Rosario Vidal, Jorge Aparicio

**Affiliations:** 1 Medical Oncology Department, Complexo Hospitalario Universitario, Ourense (CHUO), C/ Ramón Puga, 56, 32005 Ourense, Spain; 2 Medical Oncology Department, Hospital Universitario Miguel Servet, Saragossa, Spain; 3 Medical Oncology Department, Hospital Universitario Reina Sofía, Córdoba, Spain; 4 Medical Oncology Department, Hospital Universitario Vall D’Hebron, Barcelona, Spain; 5 Medical Oncology Department, Hospital General Universitario Gregorio Marañón, Madrid, Spain; 6 Medical Oncology Department, Hospital Universitario 12 de Octubre, Madrid, Spain; 7 Medical Oncology Department, Hospital Clínic, Barcelona, Spain; 8 Medical Oncology Department, Hospital Universitario de Navarra, Pamplona, Spain; 9 Medical Oncology Department, Complejo Asistencial Universitario, Salamanca, Spain; 10 Medical Oncology Department, Hospital Universitari I Politècnic la Fe, Valencia, Spain

**Keywords:** Colorectal cancer, Metastatic disease, Systemic treatment, Guidelines

## Abstract

Colorectal cancer (CRC) is the second leading cause of cancer deaths in Spain. Metastatic disease is present in 15–30% of patients at diagnosis and up to 20–50% of those with initially localized disease eventually develop metastases. Recent scientific knowledge acknowledges that this is a clinically and biologically heterogeneous disease. As treatment options increase, prognosis for individuals with metastatic disease has steadily improved over recent decades. Disease management should be discussed among experienced, multidisciplinary teams to select the most appropriate systemic treatment (chemotherapy and targeted agents) and to integrate surgical or ablative procedures, when indicated. Clinical presentation, tumor sidedness, molecular profile, disease extension, comorbidities, and patient preferences are key factors when designing a customized treatment plan. These guidelines seek to provide succinct recommendations for managing metastatic CRC.

## Incidence and epidemiology

Colorectal cancer (CRC) has the third highest incidence of all cancers worldwide, with 2022 estimates of 1,931,590 cases (10.7%), and the second highest mortality, with 935,173 deaths (9.4%) in 2020 [1]. It has been estimated that it will be the most frequently diagnosed tumor in Spain, with 43,370 new cases in 2022. In 2020, the estimated prevalence at 5 years was 191,884 cases. CRC was responsible for 11,131 deaths in 2020, with an expected increase to 17,735 by 2040 [2]. Approximately 20% of patients with CRC have metastases at the time of diagnosis, while up to 50% of those whose disease was initially localized will develop metastases. More often than not, metastatic disease is non-curable and median survival times do not exceed 30 months.

Most cases are sporadic (75–80%) and approximately 20% present familial aggregation. Only 5–7% cases are due to germline deleterious genetic variants that cause known hereditary diseases, such as Lynch syndrome and familial adenomatous polyposis [3]. Of the risk factors for sporadic CRC, aging is the most important one and has been growing exponentially through the years. Other related factors include inflammatory bowel disease and environmental factors, some of which are modifiable, such as obesity, sedentary lifestyle, diet rich in red and/or processed meats and low in fiber, and alcohol and tobacco use [4].

## Methodology

This guideline is based on a systematic review of relevant published studies and with the consensus of ten oncologist experts in treatment from two Spanish digestive cooperative groups (Grupo Español Multidisciplinar de Cáncer Digestivo, GEMCAD, and Grupo Español de Tumores Digestivos, TTD), the Spanish Society of Medical Oncology (SEOM), and an external review panel comprising two experts designated by SEOM. The Infectious Diseases Society of America–US Public Health Service Grading System for Ranking Recommendations in Clinical Guidelines has been used to assign levels of evidence and grades of recommendation [5] (Table 1).

**Table 1:** Levels of evidence and grades of recommendation

Levels of evidence
I. Evidence from at least one large randomized, controlled trial of sound methodological quality (low potential for bias) or meta-analyses of well-conducted randomized trials without heterogeneity
II. Small randomized trials or large randomized trials with a suspicion of bias (lower methodological quality) or meta-analyses of such trials or of trials with proven heterogeneity
III. Prospective cohort studies
IV. Retrospective cohort studies or case–control studies
V. Studies without control group, case reports, experts opinions
Grades of recommendation
a. Strong evidence for efficacy with a substantial clinical benefit; strongly recommended
b. Strong or moderate evidence for efficacy, but with limited clinical benefit; generally recommended
c. Insufficient evidence of efficacy or benefit does not outweigh the risk or the disadvantages (adverse events, costs,); optional
d. Moderate evidence against efficacy or for adverse outcome; generally not recommended
e. Strong evidence against efficacy or for adverse outcome; never recommended

## Diagnosis and staging

Upon suspicion of CRC based on suggestive symptoms or screening tests, a complete colonoscopy with biopsy to locate the primary tumor and confirm the pathological diagnosis is mandatory. Virtual colonoscopy is an alternative to detect potential synchronous colorectal lesions if a full colonoscopy is not feasible [I, A] [6, 7].

Diagnostic procedures should include a complete medical history (comorbidities, previous oncologic treatments, and family history of cancer), symptoms related to disease, as well as performance status, clinical examination, and laboratory tests (liver and renal function, blood count, and serum carcinoembryonic antigen) [8].

Computed tomography (CT) scan of the chest, abdomen, and pelvis is the best technique to assess distant extent [IV, A]. Magnetic resonance imaging (MRI) of the liver might be considered in certain cases, such as resectable or potentially resectable hepatic metastases [IV, A], evaluation of locally advanced tumors (especially rectal cancer), or for patients with iodine-contrast allergies, chronic kidney disease, or hepatic steatosis. A 18 F-fluorodeoxyglucose positron emission tomography (PET–CT) scan is not routinely recommended, but should be contemplated in selected subjects with increased tumor markers without evidence of metastatic disease or those with oligo-metastatic diseases that are potentially curable with local treatments [IV, B] [9].

To optimize the treatment strategy, individuals with metastatic colorectal cancer (mCRC) should be evaluated by an experienced multidisciplinary team at diagnosis and later, if necessary [III, A] [10]. The recommended staging system is that of the 8th edition of the American Join Committee on Cancer (AJCC) [I, A] [11].

### Recommendations

- A complete colonoscopy with biopsy to confirm the diagnosis is mandatory. Virtual colonoscopy is an alternative to detect potential synchronous colorectal lesions if a full colonoscopy is not feasible [I, A].
- CT scan of the chest, abdomen, and pelvis is the best technique to assess distant metastases [IV, A].
- MRI and PET-CT may be considered in selected cases [IV, B].
- Patients with mCRC should be evaluated by a multidisciplinary team to define patient management: resectable, potentially resectable, and un-resectable disease [III, A].
- The recommended staging system is that of the 8th edition of the AJCC [I, A].

## Biomarkers

Molecular profiling of mCRC and identification of specific biomarkers and molecular targets can serve as predictive and prognostic indicators of disease and response to targeted therapies. When incorporated into clinical decision-making, biomarkers and molecular targets are critical tools in personalizing therapy.

Testing for RAS (KRAS/NRAS) and BRAF mutations, as well as high microsatellite instability (MSI-H) or deficient mismatch repair proteins (dMMR) is recommended in all cases at the time of mCRC diagnosis [I, A] [12–14]. Liquid biopsy can be contemplated for molecular profiling when conventional tumor biopsy is not available or to monitor emergent mutations of resistance to targeted therapy, especially prior to re-challenge with anti-epidermal growth factor receptor (anti-EGFR) treatment, although this has yet to be approved by our national authorities [II, B] [15].

In pivotal randomized clinical trials in mCRC, the role of KRAS and NRAS (exons 2, 3, and 4) hotspot mutations has been demonstrated as negative predictive factors for response to anti-EGFR monoclonal antibodies (MoAbs) [12, 16–19]. Beyond these widely approved biomarkers, primary tumor location has been reported to be predictive of response to these therapies in the front-line setting of all RAS/BRAF wild-type tumors. In two pooled analyses including six randomized clinical trials, participants with left-sided tumors exhibited better overall survival, progression-free survival, and response rate to first-line chemotherapy combinations plus anti-EGFR agents, whereas individuals with right-sided tumors benefited more from standard chemotherapy + / − bevacizumab [20–22].

BRAF V600E mutations have proven to confer poor prognosis in advanced disease, hence the recommendation to detect their presence/absence together with RAS mutations. Moreover, the BRAF V600E mutation comprises a positive predictive factor for response to dual BRAF/EGFR inhibition in second and third line (encorafenib/cetuximab) [23].

MSI-H or dMMR must be assessed by PCR and/or immunohistochemistry (IHC) (MLH1, MSH2, MSH6, and PMS2), respectively [16], to assist clinicians with genetic counseling, including identifying Lynch syndrome [II, B], and selecting patients for immune checkpoint inhibition (ICI) [I, A] [13, 14].

HER2 overexpression by IHC or amplification by fluorescence in situ hybridization (FISH) has yet to prove its role as a poor prognosis biomarker. Several dual blockade HER2-targeted therapies have exhibited significant efficacy, although they have yet to be approved by our national authorities. Consequently, knowledge about this (covered by a prescreening research program in subsequent lines of treatment and, particularly, in RAS/BRAF wild-type populations) can contribute to determining eligibility to participate in clinical trials of these compounds [III, C] [24].

NTRK fusions constitute an uncommon molecular event in CRC, confined to RAS and BRAF wild-type tumors, predominantly in MSI-H/dMMR. This subset of patients would be eligible for undergoing the test in subsequent lines of treatment to consider accessibility to clinical trials [III, A] [25].

Regulatory agencies recommend di-hydro-pyridine dehydrogenase (DPYD) genotyping or phenotyping [III, A]. DPYD gene variants can lead to severe toxicities with fluoro-pyrimidines. Individuals harboring these alterations should receive lower doses of these compounds or even skip them for alternative regimens [26]

Next-generation sequencing (NGS) platforms have not been universally established in our country for mCRC molecular studies and are useful tools to analyze RAS, BRAF, and HER2 alterations simultaneously and providing information regarding tumor hyper-mutation burden (TMB), as well as complementary diagnostic information about Lynch syndrome by mutational study of the MMR and EPCAM genes [27].

Table 2 illustrates the staging procedures and standard biomarkers suggested for all cases of mCRC.

**Table 2:** Suggested staging procedures and standard biomarkers

Clinical examination
Laboratory tests including liver and renal function tests
Carcinoembryonic Antigen (CEA)
Pathological examination of a tumor biopsy (histological subtype, tumor grade, microsatellite status, and KRAS, NRAS and BRAF mutational status)
DPYD genotyping or phenotyping
Computed tomography (CT) scan of the chest, abdomen, and pelvis
Magnetic resonance imaging (MRI) could be considered in cases of hepatic metastases and locally advanced rectal tumors
Complete colonoscopy to locate the primary tumor, obtain tissue for histological diagnosis, and detect potential synchronous colorectal lesions
Virtual colonoscopy can be of use for tumors that prevent the endoscopic tube from advancing further
Needle biopsy of a tumor with known histologic diagnosis is only recommended when it may change the treatment strategy
Abdominal MRI with intravenous contrast may be considered if liver-directed therapy or surgery is contemplated, and for patients allergic to iodine
A fluorodeoxyglucose (FDG)-positron emission tomography (PET-CT) scan should be performed in subjects with potentially surgically curable metastatic disease

### Recommendations

- KRAS, NRAS exons 2, 3, and 4, and BRAF V600E mutations should be tested at the time of mCRC diagnosis [I, A].
- Assessment of mismatch repair deficiency (IHC or MSI-H) is recommended to assist genetic counseling for Lynch syndrome [II, B] and for its predictive value of benefit from ICI [I, A].
- Identification of HER 2 amplification or overexpression [III, C] and NTRK fusions are recommended in subsequent lines for access to clinical trials with targeted therapies [III, A].
- Liquid biopsy might be considered to monitor emergent mutations of resistance to targeted therapy, especially prior to re-challenge with anti-epidermal growth factor receptor (anti-EGFR) treatment, though this is not supported by our national authorities [II, B].
- Testing for DPYD deficiency is strongly recommended prior to initiating 5-fluorouracil-based chemotherapy [III, A].

## Resectable disease

At diagnosis, mCRC may be technically resectable in a small percentage of patients. It is considered a potentially curable disease, with reported 5-year overall survival (OS) rates of 20–45% in a highly selected population [28]. Commonly involved sites are liver, lung, peritoneum, lymph nodes, and ovary. Resection should not be undertaken unless complete removal of all known tumor and metastatic sites (R0 resection) is feasible, because incomplete resection or de-bulking has failed to prove to prolong OS.

Resectable CRC liver metastases are defined as metastatic disease amenable to R0 resection while leaving at least 20–25% of total liver volume as future organ remnant, with adequate vascular inflow and outflow, and sufficient biliary drainage [28]. Certain preoperative factors are independent predictors of poor survival: T4 primary tumor, synchronous metastatic presentation, ≥ 4 liver metastases, diameter ≥ 5 cm in the largest liver metastasis, and serum CEA level ≥ 5 ng/ml. Based on the afore-mentioned factors, resectable cases can be divided into high risk (≥ 3 factors) and low risk (fewer than 3 factors) [10]. Other poor prognostic factors are the presence of extrahepatic metastases, > 3 cm of the primary tumor diameter, and disease-free interval from diagnosis of localized to metastatic disease < 12 months [29, 30].

Extrahepatic disease is not regarded as an absolute contraindication to surgery and resection may lead to significant OS benefit in well-selected patients [31, 32]. Ablative techniques, such as stereotactic radiotherapy (SBRT) and thermal ablation, may be contemplated alone or in conjunction with resection [10].

In subjects with resectable disease and favorable prognostic criteria, perioperative treatment may not be necessary and upfront R0 resection is recommended [III, B]. In contrast, when prognosis is unclear or unfavorable, perioperative combination chemotherapy should be pondered over [II, B] [33, 34]. The preferred treatment should be FOLFOX (or alternatively CAPOX) [II, B] [33]. EGFR-targeting antibodies (MoAbs) are not recommended in this setting [IV, B] [35]. No data with bevacizumab are available for this specific group. Adjuvant chemotherapy is not recommended for individuals with favorable oncological and technical (surgical) criteria who have not received perioperative chemotherapy [II, C], but may be beneficial for those with unfavorable criteria [IV, D] [36]. Decision-making should therefore include patient characteristics and preferences. The most recent randomized clinical phase II/III trial demonstrated that postoperative chemotherapy with mFOLFOX6 improved disease-free survival (DFS), yet failed to impact OS [37].

The treatment recommendations above may also apply to pulmonary mCRC, albeit with less evidence. Complete resection is required, while maintaining adequate function. Ablative techniques may also be considered alone or in conjunction with resection. Surgery of pulmonary metastases has achieved a 3-year recurrence-free survival in 28% and 3-year OS in 78% of the cases [38].

In subjects with limited peritoneal carcinomatosis, complete cyto-reductive surgery may provide prolonged survival when performed in high-volume centers [II, A] [39]. However, the addition of hyperthermic intraperitoneal chemotherapy (HIPEC) has not proven beneficial in randomized trials, and, therefore, cannot be recommended as a standard of care [IV, B] [40].

**Fig. 1:**
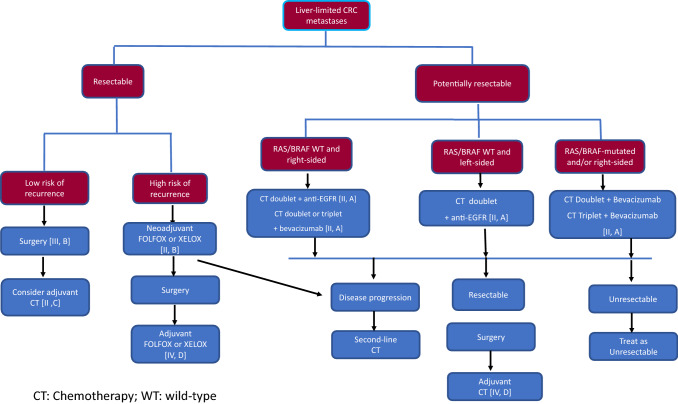
Resectable and potentially resectable mCRC

Fig. 1 illustrates the suggested treatment algorithm for individuals with mCRC limited to the liver.

### Recommendations

- Upfront resection is recommended for resectable hepatic or lung metastases with favorable prognostic criteria [III, B].
- In resectable metastases with unfavorable prognostic factors, perioperative/adjuvant therapy with FOLFOX/CAPOX should be considered [II, B].
- Complete cyto-reductive surgery should be performed in experienced centers [II, A]. However, the addition of HIPEC is not recommended [IV, B].

## Potentially resectable disease

Conversion chemotherapy refers to patients initially diagnosed with un-resectable disease, in whom an optimal response to chemotherapy would enable resection of metastatic sites. In the case of liver resection, salvage surgery is estimated to be feasible in 12.5% of these patients following chemotherapy with or without targeted agents [41]. Potentially resectable cases are defined as those who present the following situations:

- Post-resection liver remanent (LR) is inadequate either in volume or quality. At least 25% is the minimum safe LR volume needed after hepatic resection in individuals with a normal liver, and 40% if sinusoidal obstruction syndrome, cholestasic, steatosic, or cirrhotic liver is present. Treatment focuses on increasing LR volume and enhancing function, and strategies such as percutaneous transhepatic portal vein embolization should be discussed [42].
- A large tumor burden compromising R0 resection.

Such cases must be down-staged to obtain negative margin resection and neoadjuvant systemic treatment and/or locoregional therapies may be needed. The objective of systemic treatment is to decrease tumor size, prevent disease progression, and eradicate any remnant of disease. Treatment response must be closely monitored every 2 months so that resection can be undertaken as soon as the metastases become resectable, avoiding unnecessary liver toxicity [III, A]. RECIST criterion, based on the metastatic size, is the standard method to evaluate treatment response. However, if treatment with anti-vascular endothelial growth factor (VEGF) MoAbs are administered, morphological response criteria for CT imaging are preferred [43].

Chemotherapy schedules are recommended with high response rates (RR) or with a short time to response. Furthermore, adding targeted agents to a cytotoxic doublet or triplet should be contemplated according to tumor side and molecular profile.

In a systematic review of patients with liver only disease treated with chemotherapy and a targeted agents, R0 resection rates of 27–52% and median OS of 20–49 months were reported. Triplet therapy demonstrated benefit in RR and R0 resection rates compared to doublets, and the addition of bevacizumab to a triplet regimen displayed higher RR and prolonged progression-free survival (PFS) [41]. RAS mutational status was found to be the most important predictive feature for conversion chemotherapy. In RAS wild-type patients, the addition of anti-EGFR agents to a cytotoxic doublet was associated with significantly higher RR, R0 resection rates, and OS [44].

Data from a retrospective pooled analysis of six trials found that patients with left-sided RAS wild-type tumors receiving chemotherapy plus EGFR MoAb therapy had higher RR, PFS, and OS than patients treated with chemotherapy alone or with chemotherapy plus bevacizumab; consequently, this therapy is recommended [II, A]. In subjects with RAS wild-type right-sided tumors, treatment with chemotherapy and anti-EGFR MoAb also exhibited higher RR, as did triplet therapy with bevacizumab. Both combinations are therefore recommended [20, 45] [II, A].

In cases of RAS mutant, un-resectable CRC liver-limited metastases, bevacizumab combined with cytotoxic doublets or triplets increased the resectability rate and improved RR and OS compared chemotherapy alone [45].

In the setting of un-resectable mCRC, transarterial radioembolization (TARE) with microspheres impregnated with yttrium-90 (Y90) together with chemotherapy was associated with a statistically significant increase in the potentially curative resectability of the liver [IV, D] [46]. Hepatic arterial infusion chemotherapy has emerged as a complementary method to systemic chemotherapy in conversion strategy [III, B] [47]. Oncosurgical strategies, such as two-stage hepatectomy (TSH) [48], a combination of surgical resection with local ablation, or ALPPS procedure (associating liver partition and portal vein ligation for stage hepatectomy) [49] may help un-resectable metastases become resectables.

### Recommendations

- A regimen leading to high RR and/or making a large tumor smaller is recommended for individuals with potentially resectable CRC liver metastases [II, A].
- Response to treatment must be closely monitored every 2 months [III, A].
- A cytotoxic doublet plus an anti-EGFR is recommended in cases of RAS wild-type disease and left-sided tumors [II, A].
- For patients with RAS wild-type disease and right-sided tumors, a cytotoxic doublet plus an anti-EGFR antibody or a cytotoxic doublet or triplet plus or minus bevacizumab is recommended [II, A].
- For subjects with RAS mutant disease, a cytotoxic doublet or triplet plus or minus bevacizumab is recommended [II, A].

## Unresectable disease: First-line therapy

The cornerstone of first-line therapy for un-resectable metastases from CRC is systemic treatment. Surgery of primary tumor should only be considered in symptomatic patients. There is no evidence of increased OS as a result of resecting an asymptomatic primary tumor in cases of synchronous un-resectable metastases [II, B] [50].

The foremost treatment endpoints in this setting include prolonging survival, relieving symptoms secondary to the disease, and improving and maintaining quality of life. The most relevant factors that bear upon treatment selection (in all lines of therapy) are related to tumor (molecular profile, tumor burden, related symptoms, primary tumor sidedness, and progression dynamics), patient (performance status, comorbidities, age, and expectations), and treatment characteristics (toxicity profile, cost, and availability) [16]. In the first-line setting, delivering chemotherapy in combination with biological therapy (anti-VEGF) or anti-EGFR MoAb) is generally recommended.

Based on their general condition, patients are classified as fit or unfit. Unfit individuals must be defined by patient-related characteristics and not by tumor-derived symptoms. Clinical guidelines recommend some type of therapy in these cases (not eligible for intensive therapy [51] or potentially suitable to receive treatment) [16]. For unfit subjects with wild-type RAS/BRAF and left-sided tumors, monotherapy with anti-EGFR MoAb [IV, C] [52] or, preferably a combination of 5-fluorouracil with anti-EGFR [I, A] or 5-fluorouracil ± bevacizumab [I, B] [53, 54] are well tolerated and have displayed efficacy, whereas in unfit patients with RAS/BRAF mutation or right-sided tumors, fluoropyrimidine ± bevacizumab is the most appropriate combination [I, B] [54]. Similarly, monotherapy with fluoropyrimidine can be contemplated in individuals with certain comorbidities.

For MSI-H/dMMR tumors, ICI is the treatment of choice. Pembrolizumab has been shown to increase RR and PFS compared to chemotherapy combinations. Moreover, there was less severe toxicity with ICI. No differences in OS could be demonstrated, inasmuch as 60% of the individuals who received chemotherapy were crossed over to pembrolizumab [I, A] [13].

**Fig. 2:**
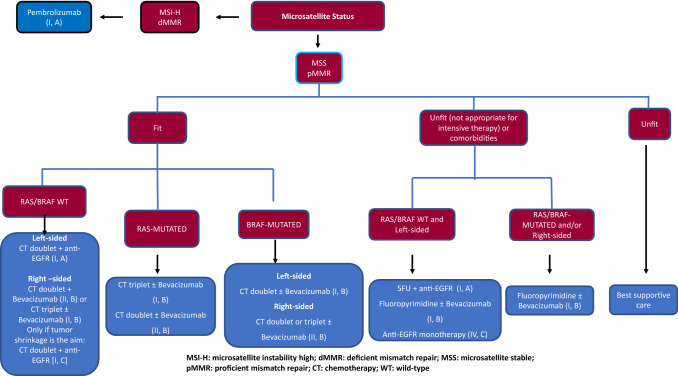
Unresectable mCRC. First-line therapy

For fit subjects with microsatellite stable disease, first-line treatment consists of a chemotherapy doublet consisting of the anti-VEGF MoAbs bevacizumab or anti-EGFR agents, such as cetuximab or panitumumab (Fig. 2). Chemotherapy combinations include doublets of fluoropyrimidine (5-fluorouracil or capecitabine) plus oxaliplatin or irinotecan (FOLFOX, FOLFIRI, CAPOX). The treatment recommendation for wild-type RAS and BRAF tumors is based on primary tumor location. RR and OS are better for people with left-sided tumors who receive a combination of chemotherapy with anti-EGFR MoAb [I, A]. In contrast, for patients with right-sided tumors, a greater OS and PFS benefit has been suggested for chemotherapy alone or combined with bevacizumab [II, B] [21, 55, 56]. For RAS or BRAF-mutated tumors, the first-line treatment recommendation is chemotherapy combination (doublet or triplet) with bevacizumab [II, B]. In one meta-analysis, the chemotherapy triplet of 5-fluorouracil, oxaliplatin, and irinotecan together with bevacizumab has exhibited improved OS, PFS, and RR, with respect to doublets + bevacizumab, mainly in RAS-mutated and right-sided tumors, with a moderate increase in toxicity. The benefit of this combination is limited to selected patients (ECOG 0–1, < 70–75 years old, with no comorbidities and no previous oxaliplatin-based adjuvant chemotherapy) [I, B] [57]. For patients with wild-type RAS/BRAF, triplet in combination with an anti-EGFR mAb has proven no benefit in terms of treatment activity and increased gastrointestinal toxicity and, therefore, cannot be recommended [II, E] [58].

Recommendations concerning the duration of first-line therapy in mCRC have evolved in recent years. Historically, the disease was treated continuously until progression, unacceptable toxicity, or the patient’s wish. Currently, treatment duration is subjected to the person’s personal circumstances, cumulative treatment toxicity, response to first-line treatment, and the aggressiveness of the disease. Response should be assessed every 8–12 weeks, with treatment de-escalation allowed after 4–6 months (mainly in those patients receiving oxaliplatin) to prevent the appearance of irreversible sensory neurotoxicity. Hence, different strategies have emerged including stop-and-go or intermittent therapy, as well as maintenance treatment. After induction chemotherapy combined with bevacizumab, fluoropyrimidine maintenance with or without bevacizumab significantly improves PFS and reveals a trend toward prolonged OS and, hence, should be considered [I, B] [59]. Randomized phase II studies have shown an increase in efficacy with 5-fluorouracil with anti-EGFR MoAb maintenance *versus* anti-EGFR or 5-fluorouracil in monotherapy (II, B) [60].

### Recommendations

- Resection of an asymptomatic primary tumor is not recommended [II, B].
- For unfit patients with wild-type RAS/BRAF and left-sided tumors, monotherapy with anti-EGFR agents [IV, C] or, preferably a combination of 5-fluorouracil with anti-EGFR [I, A] is recommended.
- For unfit subjects with mutated RAS/BRAF and/or right-sided tumors, fluoropyrimidine ± bevacizumab is the most suitable combination [II, B].
- In cases of MSI-H/dMMR tumors, ICI (pembrolizumab) is the treatment of choice [I, A].
- A combination of chemotherapy doublet with anti-EGFR MoAb is recommended for wild-type RAS and BRAF and left-sided tumors [I, A]. Triplets plus anti-EGFR are not deemed appropriate in this context [II, E].
- For patients with mutated RAS or BRAF and/or right-sided tumors, chemotherapy doublets with or without bevacizumab are recommended [II, B]. Triplets with bevacizumab should be considered in selected cases (ECOG 0–1, < 70–75 years old and no previous oxaliplatin-based adjuvant chemotherapy) [I, B].
- After induction chemotherapy with bevacizumab, fluoropyrimidine with or without bevacizumab maintenance therapy should be considered [I, B].
- After induction treatment with chemotherapy plus anti-EFR MoAbs, maintenance with fluropyrimidine plus anti-EGFR may also be contemplated [II, B]

## Unresectable disease: Second-line

Almost all individuals treated with first-line therapy who do not benefit from curative treatment strategies will progress and more than 60% of them can be considered for second-line treatment. Patients should receive all available treatments during the course of the disease. Given the palliative nature of this therapy and its modest efficacy and toxicity profile, physician–patient shared decision-making is recommended. Selected cases of indolent disease and comorbidities can be managed, at least temporally, without oncological treatment.

**Fig. 3:**
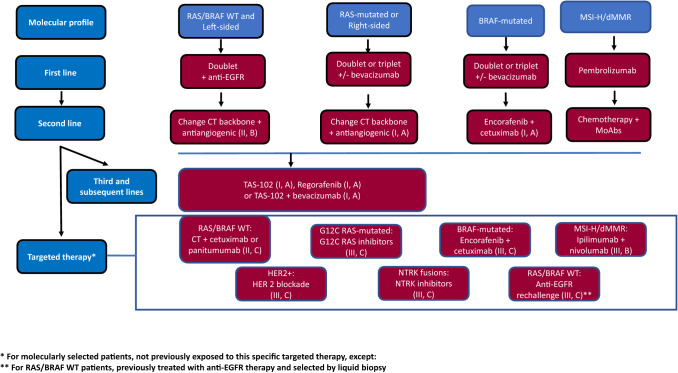
Unresectable mCRC. Second and successive lines of therapy

Second-line strategy will depend on prior treatment and patient preferences. For subjects who received first-line oxaliplatin-based therapy, FOLFIRI and irinotecan alone are the preferred options. When the previous treatment was an irinotecan-based combination, the recommended options are FOLFOX and CAPOX. Recommendations on targeted therapies are based on RAS and BRAF status (Fig. 3).

For RAS-mutated tumors, bevacizumab and aflibercept added to chemotherapy are both options in second-line therapy [I, A] [61, 62]. In cases previously treated with first-line bevacizumab-containing chemotherapy, the continuation of bevacizumab in conjunction with a second-line chemotherapy improves OS compared to simply switching the chemotherapy regimen alone [63]. In the BRAF-mutated setting, the combination of encorafenib and cetuximab has exhibited increased efficacy in terms of RR, PFS, and OS with respect to cetuximab–irinotecan [I, A] [23]. Efforts should be dedicated to identifying biomarkers that could improve efficacy in this devastating subset of tumors. In double wild-type patients (RAS and BRAF WT), the standard therapy is to change the doublet and, in those not previously treated with anti-EGFR, MoAbs should be contemplated [II, C] [64, 65]. In the context of treatment with first-line chemotherapy and anti-EGFR MoAbs, the addition of bevacizumab or aflibercept in second-line is not born out of phase III clinical trials. In dMMR/MSI-H tumors progressing after first-line chemotherapy, ipilimumab plus nivolumab or pembrolizumab is recommended [66] [III, B].

### Recommendations

- For RAS-mutated neoplasms, bevacizumab and aflibercept added to chemotherapy are options in second-line therapy [I, A].
- In BRAF-mutated tumors, the combination of encorafenib and cetuximab is recommended. However, these treatments are not yet approved by the Spanish healthcare authorities. [I, A].
- The standard second-line therapy for RAS and BRAF WT is doublet crossover and, in patients not previously treated with anti-EGFR MoAbs, the addition of cetuximab or panitumumab [II, C].
- For dMMR/MSI-H tumors progressing after first-line treatment in RAS and BRAF WT patients, line chemotherapy, ipilimumab plus nivolumab or pembrolizumab is recommended [III, B].

## Unresectable disease: third and successive line

Classically, 30–40% of patients with mCRC were eligible to receive third-line therapy. However, this percentage has steadily increased as more treatment options have become available. In this setting, targeted therapies can be classified as histology-tuned (RAS, BRAF, and HER2) and histology-agnostic (MSI and NTRK). Subjects with specific molecular alterations (i.e., RAS WT, BRAF V600E mutant or MSI-H/dMMR) and not exposed to targeted therapy in prior lines (anti-EGFR, anti-BRAF + anti-EGFR, or immunotherapy, respectively), should receive them in third line. Other directed therapies are still deemed experimental and would be preferably used in the setting of clinical trials (Fig. 3).

Trifluridine–tipiracil (TAS-102) and regorafenib are two oral drugs approved in refractory mCRC given their limited, yet statistically significant benefits on PFS and OS as compared to placebo in their respective phase III trials [I, A] [67–70].

TAS-102 is an oral fluoro-pyrimidine with a favorable toxicity profile. The most common adverse events are neutropenia and leukopenia, although febrile neutropenia is uncommon (< 5%). Neutropenia during the first two courses of treatment has been suggested as a positive predictive factor for response [67, 68]. Recently, a beneficial effect on OS, PFS, and RR, and time to worsening to an ECOG PS of > 2 have been reported for the combination of TAS-102 + bevacizumab *versus* TAS-102 alone in a phase III trial and should be recommended in the third-line setting, albeit approval for its use in our national healthcare system is pending [I, A] [71]. Regorafenib is a multikinase inhibitor with antiangiogenic, anti-stromal, and anti-tumor activity. The most common grade ≥ 3 adverse events are hand–foot syndrome, hypertension, fatigue, and diarrhea [69, 70]. To overcome tolerance issues, various treatment schedules and dosage modifications have been proposed [72].

Fruquintinib is another oral multikinase inhibitor, already approved in China for refractory mCRC. The final results of the FRESCO-2phase III trial (with both occidental and Asian populations) were presented at ESMO 2022. Median OS and PFS were significantly better with respect to placebo. Most participants in this trial had received prior TAS-102 and/or regorafenib [73].

Re-challenge with anti-EGFR agents is an option for tumors that have responded or stabilized with prior anti-EGFR therapy [74]. In phase II trials, third-line RR is 20–30%, with median PFS of 3–4.5 months, and OS of 5–12 months [III, C]. Detection of mutations in ctDNA by liquid biopsy is key to selecting individuals who are most amenable to this treatment approach, though it has not been approved in our country [75].

Some 3–4% of patients with mCRC present HER2 amplification/overexpression and should be considered for treatment with anti-HER2 targeted agents, despite not having been approved by our national healthcare system. Trastuzumab should be combined with a second HER2 inhibitor (lapatinib, pertuzumab) to achieve significant activity [III, C]. The efficacy of these double inhibition or the antibody–drug conjugate (trastuzumab–deruxtecan) has been demonstrated in phase II trials (ORR 30–52% and median PFS 2.9–8.1 months). [24, 76].

KRAS G^12C^ mutations are detected in 3–4% of mCRC patients. Two oral selective inhibitors (sotorasib and adagrasib) are being tested both in monotherapy and in combination with anti-EGFR MoAbs with promising early results. [77, 78]. Finally, NTRK fusions are present in < 2.5% of patients and enriched in right-sided tumors, MSI-H, and native RAS/BRAF tumors. The preliminary activity of targeted drugs is worth mentioning. Larotrectinib and entrectinib have been approved by the EMA and FDA as agnostics for tumors with NTRK rearrangements, but not by our national healthcare system authorities [79, 80]. ALK and ROS1 fusions are even more uncommon (< 1%) (Table 3).

**Table 3:** Summary of recommendations

Diagnosis and staging
A complete colonoscopy with biopsy to confirm the diagnosis is mandatory. Virtual colonoscopy is an alternative to detect potential synchronous colorectal lesions if a full colonoscopy is not feasible [I, A]
CT scan of the chest, abdomen, and pelvis is the best technique to assess distant metastases [IV, A]
MRI and PET-CT may be considered in selected cases [IV, A]
Patients with mCRC should be evaluated by a multidisciplinary team to define patient management: resectable/potentially resectable disease and unresectable [III, A]
Recommended staging system is that of the 8^th^ edition of the AJCC [I, A]
Biomarkers
KRAS, NRAS exon 2, 3 & 4, and BRAF V600E mutations should be tested at the time of mCRC diagnosis [I, A]
Assessment of mismatch repair deficiency (IHC or MSI-H) is recommended to assist genetic counseling for Lynch syndrome [II, B] and for its predictive value for benefit from ICI [I, A]
Identification of HER2 amplification or overexpression [III,C], and NTRK fusions are recommended in later lines to access clinical trials with targeted therapies [III, A]
Liquid biopsy may be considered to monitor emergent mutations of resistance to targeted therapy, especially prior to re-challenge with anti-epidermal growth factor receptor (anti-EGFR) treatment, though this is not supported by our national agency [II, B]
Testing for DPYD deficiency is strongly recommended before initiating 5-fluorouracil-based chemotherapy [III, A]
Resectable disease
Resection upfront is recommended for resectable hepatic or lung metastases with favorable prognostic criteria [III, B]
In resectable metastases with unfavorable prognostic factors, perioperative/adjuvant therapy with FOLFOX/CAPOX should be considered [II, B]
Complete cyto-reductive surgery should be performed in experienced centers [II, A]. However, the addition of HIPEC is not recommended [IV, B]
Potentially resectable disease
A regimen leading to high RR and/or a sizeable tumor reduction is recommended for patients with potentially resectable CRC liver metastases [II, A]
Response to treatment must be closely monitored every 2 months [III, A]
In cases of RAS wild-type disease and left-sided tumors, a cytotoxic doublet plus an anti-EGFR is recommended [II, A]
For patients with RAS wild-type disease and right-sided tumors, a cytotoxic doublet plus an anti-EGFR antibody, or a cytotoxic doublet or triplet plus or minus bevacizumab are recommended [II, A]
A cytotoxic doublet or triplet plus or minus bevacizumab is recommended for RAS mutant disease [II, A]
Unresectable disease, first-line
Resection of an asymptomatic primary tumor is not recommended [II, B]
For unfit patients with wild-type RAS/BRAF wild-type and left-sided tumors, monotherapy with anti-EGFR agents [IV, C] or, preferably a combination of 5-fluorouracil with anti-EGFR [I, A] are recommended
For unfit patients with RAS/BRAF-mutated and/or right-sided tumors, fluoropyrimidine ± bevacizumab is the most appropriate combination [II, B]
For MSI-H/dMMR tumors, ICI (pembrolizumab) represent the treatment of choice [I, A]
In cases of wild-type RAS and BRAF and left-sided tumors, a combination of chemotherapy doublet with anti-EGFR MoAb is recommended [I, A]. Triplets plus anti-EGFR are not considered appropriate in this setting [II, E]
For patients with RAS- or BRAF-mutated and/or right-sided tumors, chemotherapy doublets with or without bevacizumab are recommended [II, B]. Triplets with bevacizumab should be considered in selected patients (ECOG 0–1, < 70–75 years of age, and no previous oxaliplatin-based adjuvant chemotherapy) [I, B]
After induction chemotherapy with bevacizumab, fluoropyrimidine with or without bevacizumab maintenance therapy should be considered [I, B]
Following induction treatment with chemotherapy plus anti-EFR MoAbs, maintenance with fluoropyrimidine plus anti-EGFR may also be considered [II, B]
Unresectable disease, second line
In RAS mutant tumors, bevacizumab or aflibercept added to chemotherapy are options in second-line therapy [I, A]
In BRAF-muted neoplasms, the combination of encorafenib and cetuximab is recommended.However, these treatments are not yet approved by the Spanish healthcare authorities. [I, A]
In RAS and BRAF WT tumors, the standard second-line therapy is doublet crossover and, in patients not previously treated with anti-EGFR MoAbs, the addition of cetuximab or panitumumab is recommended [II, C]
For dMMR/MSI-H tumors progressing after first-line chemotherapy, ipilimumab plus nivolumab or pembrolizumab are recommended [III, B]
Unresectable disease, third and successive lines
TAS-102 plus bevacizumab [I, A], TAS-102 or regorafenib [I, A] are recommended in patients pre-treated with fluoro-pyrimidines, oxaliplatin, irinotecan, and biologics
Re-challenge with anti-EGFR MoAbs may be an option in selected cases having no RAS/BRAF mutations in ctDNA [III, C]
In HER2-positive patients, treatment with HER2 dual blockade is optionally recommended [III, C]

### Recommendations

- TAS-102 plus bevacizumab [I, A] and TAS-102 or regorafenib [I, A] are recommended for patients pre-treated with fluoro-pyrimidines, oxaliplatin, irinotecan, and biologics.
- Re-challenge with anti-EGFR MoAbs may be an option in selected cases with no RAS/BRAF mutations in ctDNA [III, C].
- In HER2-positive tumors, treatment with HER2 dual blockade is recommended as an option [III, C].
